# Supplementary material for: Fine-Tuning of mTORC1-ULK1-PP2A Regulatory Triangle Is Crucial for Robust Autophagic Response upon Cellular Stress
Source: Biomolecules. 2022 Oct 28;12(11):1587. doi: 10.3390/biom12111587 (PMC9687272; doi:10.3390/biom12111587)
Supplement: Supplementary file 1 [file biomolecules-12-01587-s001.zip › biomolecules-1915136-supplementary.pdf]

Supplementary Information
"Fine-tuning of mTORC1-ULK1-PP2A regulatory triangle is crucial for robust autophagic response upon cellular stress"

1 The basics of mathematical modelling

A biological regulatory network can be translated into a set of ordinary differential equation (ODE) to describe how the concentration/activity of each control element in the network changes with the time. A generic differential equation depicting the temporal changes of a regulatory element is composed of two parts: production and consumption terms. In a cellular protein-protein regulatory network the production can be given by protein synthesis (i.e., transcription and translation) and/or an activation (i.e. post-translational modification) term, while the consumption can be given by protein degradation and/or inactivation term. Usually, synthesis and degradation reactions are described by mass action kinetics, whereas protein activity can be described either by mass action or Michaelis-Menten kinetics. Solving a set of non-linear ODEs gives the time evolution of the relative protein concentration/activity (time courses).

Our model consists of six state variables, the relative concentrations of active and inactive forms of mTORC1, PP2A and ULK1 proteins. The interaction between them can be described by elementary reactions. The following reactions are included in our model:

$$mTOR_i \xrightarrow{\text{kamtor}} mTOR_a$$

$$mTOR_a \xrightarrow{\text{kimtor}} mTOR_i$$

$$mTOR_a + PP2A_a \xrightarrow{\text{kimtor2}} PP2A_a + mTOR_i$$

$$mTOR_a + ULK1_a \xrightarrow{\text{kimtor1}} ULK1_a + mTOR_i$$

$$ULK1_i \xrightarrow{\text{kaulk}} ULK1_a$$

$$ULK1_i + PP2A_a \xrightarrow{\text{kaulk1}} ULK1_a + PP2A_a$$

$$ULK1_a + mTOR_a \xrightarrow{\text{kiulk2}} mTOR_a + ULK1_i$$

$$ULK1_a \xrightarrow{\text{kiulk}} ULK1_i$$

$$PP2A_i \xrightarrow{\text{kapp2}} PP2A_a$$

$$PP2A_i + ULK1_a \xrightarrow{\text{kapp21}} PP2A_a + ULK1_a$$

$$PP2A_a \xrightarrow{\text{kipp2}} PP2A_i$$

$$PP2A_a + mTOR_a \xrightarrow{\text{kipp21}} PP2A_i + mTOR_a$$

From the elementary reactions above we can derive equations 1-6.

$$\frac{dmTOR_a}{dt} = kamtor * mTOR_i - (kimtor + kimtor1 * ULK1_a - kimtor2 * PP2A_a * mTOR_a \tag{S1}$$

$$\frac{dmTOR_i}{dt} = -\frac{dmTOR_a}{dt} \tag{S2}$$

$$\frac{dULK1_a}{dt} = kaulk * ULK1_i - kiulk * ULK1_a + kaulk1 * PP2A_a * ULK1_i - kiulk2 * mTOR_a * ULK1_a \tag{S3}$$

$$\frac{dULK1_i}{dt} = -\frac{dULK1_a}{dt} \tag{S4}$$

$$\frac{dPP2A_a}{dt} = kapp2 * PP2A_i - kipp2 * PP2A_a + kapp21 * ULK1_a * PP2A_i - kipp21 * mTOR_a * PP2A_a \tag{S5}$$

$$\frac{dPP2A_i}{dt} = -\frac{dPP2A_a}{dt} \tag{S6}$$

Post translational modifications do not change the total amount of enzymes, so we can write the following conservation laws shown in equations 7-9.

$$mTOR_T = mTOR_i + mTOR_a \tag{S7}$$

$$ULK1_T = ULK1_i + ULK1_a \tag{S8}$$

$$PP2A_T = PP2A_i + PP2A_a \tag{S9}$$

We can substitute equations 7-9 into 1-6 and we get equations 10-12.

$$\frac{dmTOR_a}{dt} = kamtor * (mTOR_T - mTOR_a) - (kimtor + kimtor1 * ULK1_a + kimtor2 * PP2A_a) * mTOR_a \tag{S10}$$

$$\frac{dULK1_a}{dt} = (kaulk + kaulk1 * PP2A_a) * (ULK1_T - ULK1_a) - (kiulk + kiulk2 * mTOR_a) * ULK1_a \tag{S11}$$

$$\frac{dPP2A_a}{dt} = (kapp2 + kapp21 * ULK1_a) * (PP2A_T - PP2A_a) - (kipp2 + kipp21 * mTOR_a) * PP2A_a \tag{S12}$$

1.1 Mathematical codes for computational simulations

All the used codes are available at `https://github.com/eraut/pp2aMtorUlk`.

1.1.1 Structural identifiability analysis

Our model has 12 unknown parameters in the form of reaction rate constants. Since their value cannot be determined through experimental methods, they has to be estimated. Before parameter estimation a structural identifiability analysis is run, which tells us if a unique parameterization exists for any given model output **?**.To determine that, Julia's StructuralIdentifiability.jl package **?** is used. If it is assumed that the inactive enzyme concentrations could be inferred from the active enzyme concentrations, then the above described model becomes structurally identifiable.

1.1.2 Parameter estimation

The final parameter values are given in Table S1. In the table, the parameter values numbered 4-6 are set to 0.15 and referred to so called "background activities". It means that they belong to effects independent from the three investigated proteins. Since both ULK1 and PP2A inhibit mTORC1 activity, the value of "kamtor" is increased and the value of "kimtor" is decreased by one order of magnitude to compensate for this effect.

The rest of the parameter values are estimated with L-BFGS-B algorithm. The cost function is defined as the mean squared deviation from the training data points. Because Western blot analysis generates data with high uncertainty, the training data set is enriched with auxiliary functions. Auxiliary functions are a cubic polynomial fitted to the measured data.

For the simulation of the rapamycin treatment equations 3-6 are used. Equations 1 and 2 are excluded since rapamycin inhibits the activity of mTORC1, therefore a constant low level of mTORC1 activity is assumed. The same logic is applied for the simulation of the okadaic acid treatment where equations 1-4 are used only, and PP2A activity is set to a constant low level.

ID	Parameter	Value $[\frac{1}{min}]$	Effect	
1	kamtor	1.5	mTORC1 autoactivation time constant	assumed
2	kimtor	0.015	mTORC1 autodeactivation time constant	assumed
3	kaulk	0.15	ULK1 autoactivation time constant	assumed
4	kiulk	0.15	ULK1 autodeactivation time constant	assumed
5	kapp2	0.15	PP2A autoactivation time constant	assumed
6	kipp2	0.15	PP2A autodeactivation time constant	assumed
7	kimtor1	5.16733842	ULK1 induced mTORC1 deactivation	estimated
8	kimtor2	0.01	PP2A induced mTORC1 deactivation	estimated
9	kaulk1	2.85833946	PP2A induced ULK1 activation	estimated
10	kiulk1	9.74873293	mTORC1 induced ULK1 deactivation	estimated
11	kapp21	14.4792405	ULK1 induced PP2A activation	estimated
12	kipp21	46.40777268	mTORC1 induced PP2A deactivation	estimated

Table S1: Parameters and their values. The last column indicates whether the given parameter got estimated by the optimization method, or it's value is assumed.

1.1.3 Model validation

The model is validated by simulating combined treatments. For this purpose, equations 10-12 are used, as the usage of these equations allow us to introduce multiple treatment at once by changing the values of constants $mTOR_T$;$ULK1_T$;$PP2A_T$. The parameter values of each combined treatment is shown in Table S2.

It is important to note that, although the model can be identified structurally theoretically, but since we have not tested a treatment in which only mTORC1 or PP2A proteins are active, the parameters obtained can be varied within a certain interval without changing the dynamics of the system.

**Treatment**	$mTOR_T$	$ULK1_T$	$PP2A_T$
rap	0.1	1	1
OA	1	1	0.1
OA + rap	0.1	1	0.1
siPP2A + rap	0.1	1	0.01
siULK1	1	0.01	1
siULK1 + rap	0.1	0.01	1
decreased ULK1 + rap	0.1	0.3	1

Table S2: Different treatments and their parameter values.OA stands for Okadaic acid, rap stands for rapamycin.

1.2 Mathematical codes for phase plane diagrams

The phase plane diagrams are computed numerically using XPP-AUT, which is freely available from `http://www.math.pitt.edu/~bard/xpp/xpp.html`. All the simulations presented in the text are based on the following XPP codes which contains ODEs. The rate constants (k) have the dimension of min-1and the proteins levels/activities are given in arbitrary units (a.u).

2 Figures

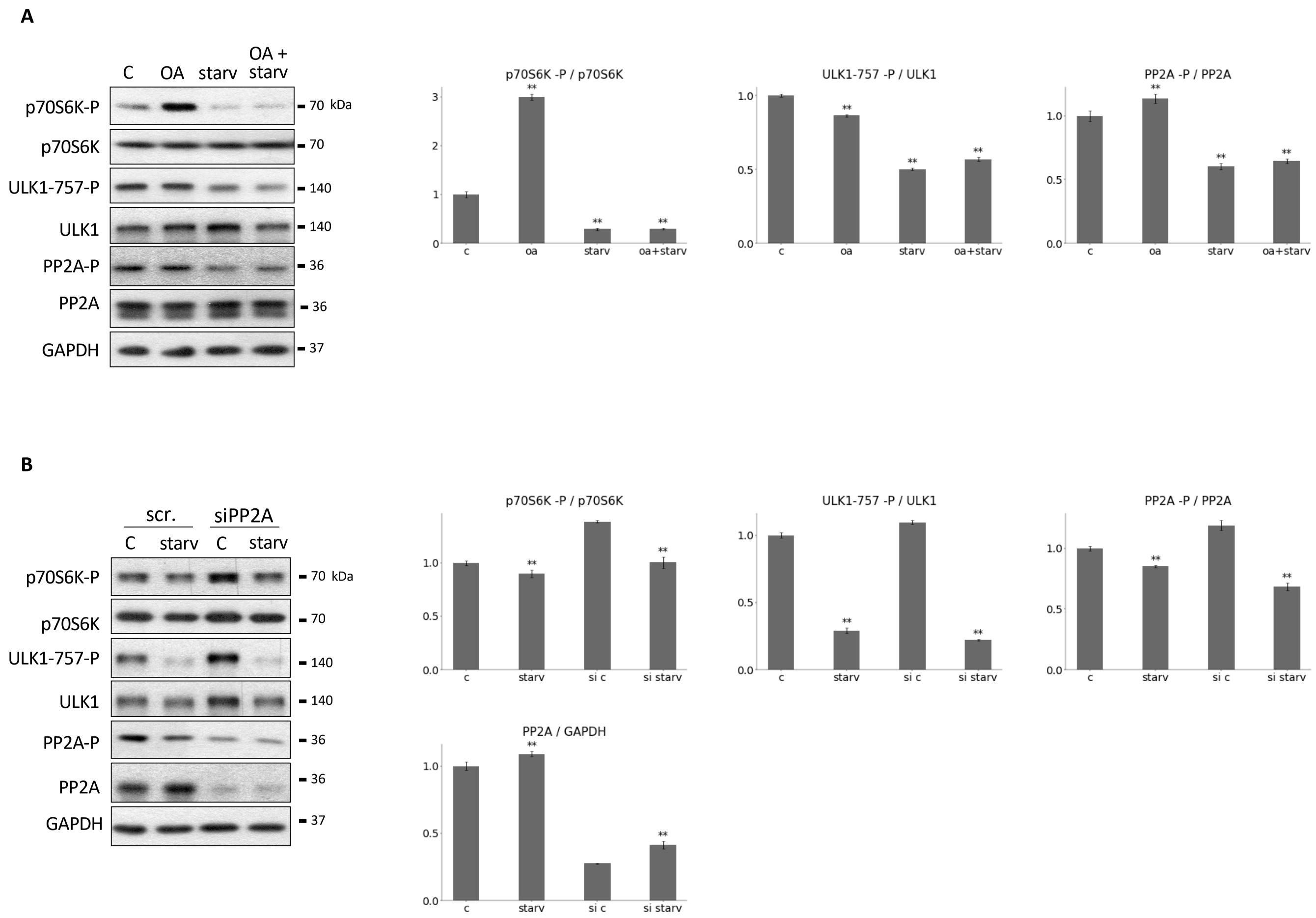

Figure S1: Combined down-regulation of both PP2A and mTORC1 can result in ULK1 activation. (A) HEK293T cells were treated with okadaic acid (OA – 3 nM, 2 h), or starved (glucose starv, 6 h), or starv + OA. (B) PP2A was silenced in HEK293T cells combined with/without starvation for 6 hours. The silencing was checked by using a scramble siRNA. (A and B, panel left) The markers of ULK1 (ULK1-757-P), PP2A (PP2A-P) and mTORC1 (p70S6K-P) were followed by immunoblotting. GAPDH was used as loading control. (A and B, panel right) Densitometry data represent the intensity of ULK1-757-P normalized for total level of ULK1, PP2A-P normalized for total level of PP2A and p70S6K-P normalized for total level of p70S6K. For each of the experiments, three independent measurements were carried out. Error bars represent standard deviation asterisks indicate statistically significant difference from the control: ns – nonsignificant; * - $p < 0.05$; ** - $p < 0.01$.

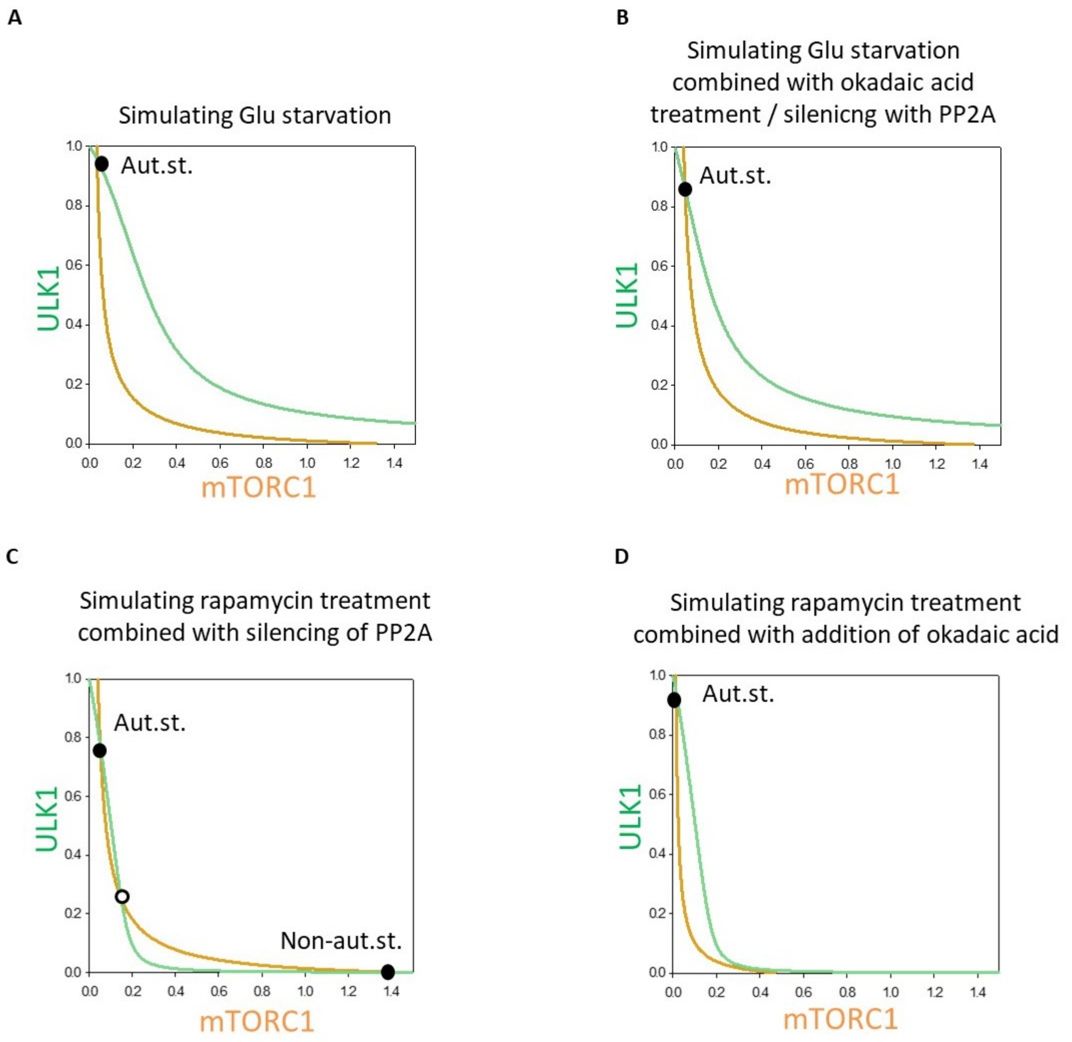

Figure S2: Theoretical analysis of combined down-regulation of both PP2A and mTOR. Phase plane diagram of PP2A-mTORC1-ULK1 regulatory triangle (A) upon starvation (starv = 0.1) or (B) starvation combined with ocadaic acid (starv = 0.1, PP2AT = 0.3) treatment, (C) upon rapamycin treatment combined with silencing of PP2A (mTORT = 0.1, PP2AT = 0.1) and (D) upon rapamycin treatment combined with okadaic acid (mTORT = 0.1, PP2AT = 0.3). The balance curves of ULK1 (green curve) and mTORC1 (red curve) are plotted. The stable and unstable steady states are visualized with black and white dots.“Non-aut.st.” refers to “non-autophagy state” (with active mTORC1 and inactive ULK1), while “Aut.st.” refers to “autophagy state” (with active ULK1 and inactive mTORC1).

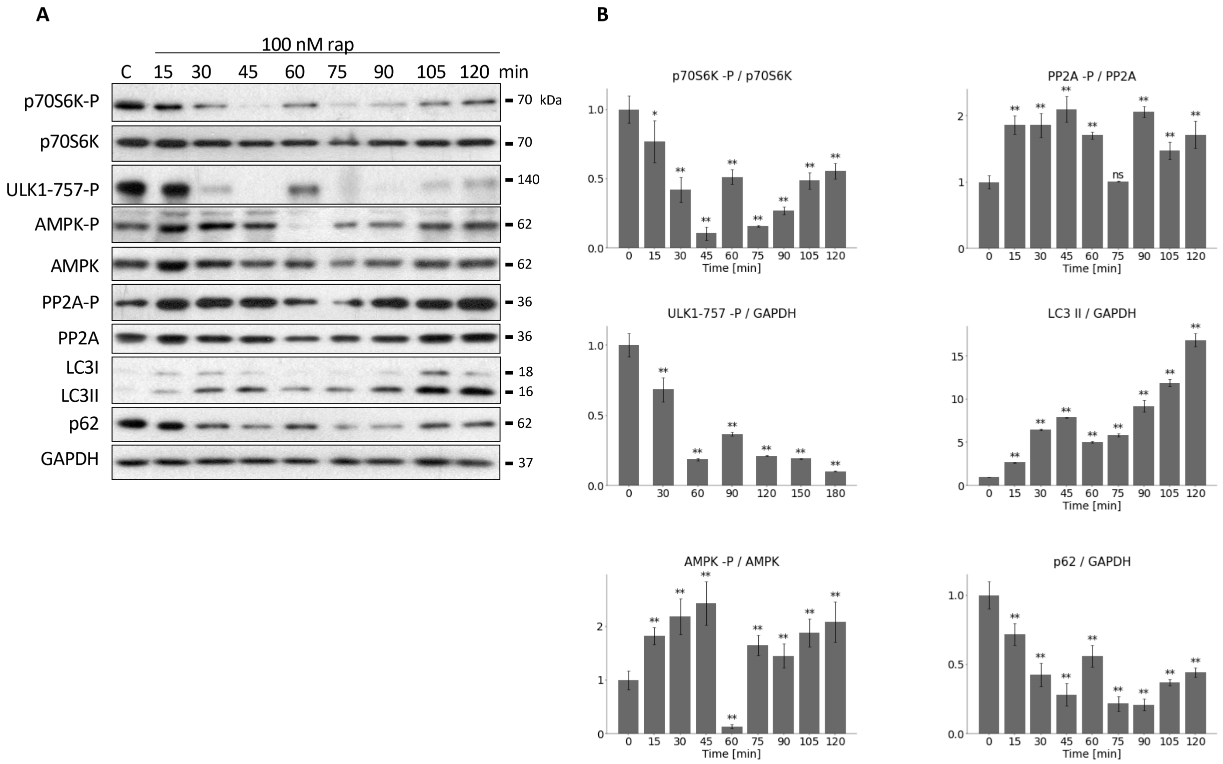

Figure S3: Prolonged rapamycin treatment results in oscillation of PP2A-mTORC1-ULK1 controlled autophagy. HEK293T cells were denoted in time after 100 nM rapamycin treatment. (A) The markers of ULK1 (ULK1-757-P), PP2A (PP2A-P), and mTORC1 (p70S6K-P), LC3 and p62 were followed by immunoblotting. GAPDH was used as loading control. (B) Densitometry data represent the intensity of ULK1-757-P normalized for GAPDH, PP2A-P normalized for total level of PP2A, p70S6K-P normalized for total level of p70S6K, LC3II and p62 normalized for GAPDH. For each of the experiments, three independent measurements were carried out. Error bars represent standard deviation asterisks indicate statistically significant difference from the control: ns – nonsignificant; * - $p < 0.05$; ** - $p < 0.01$.

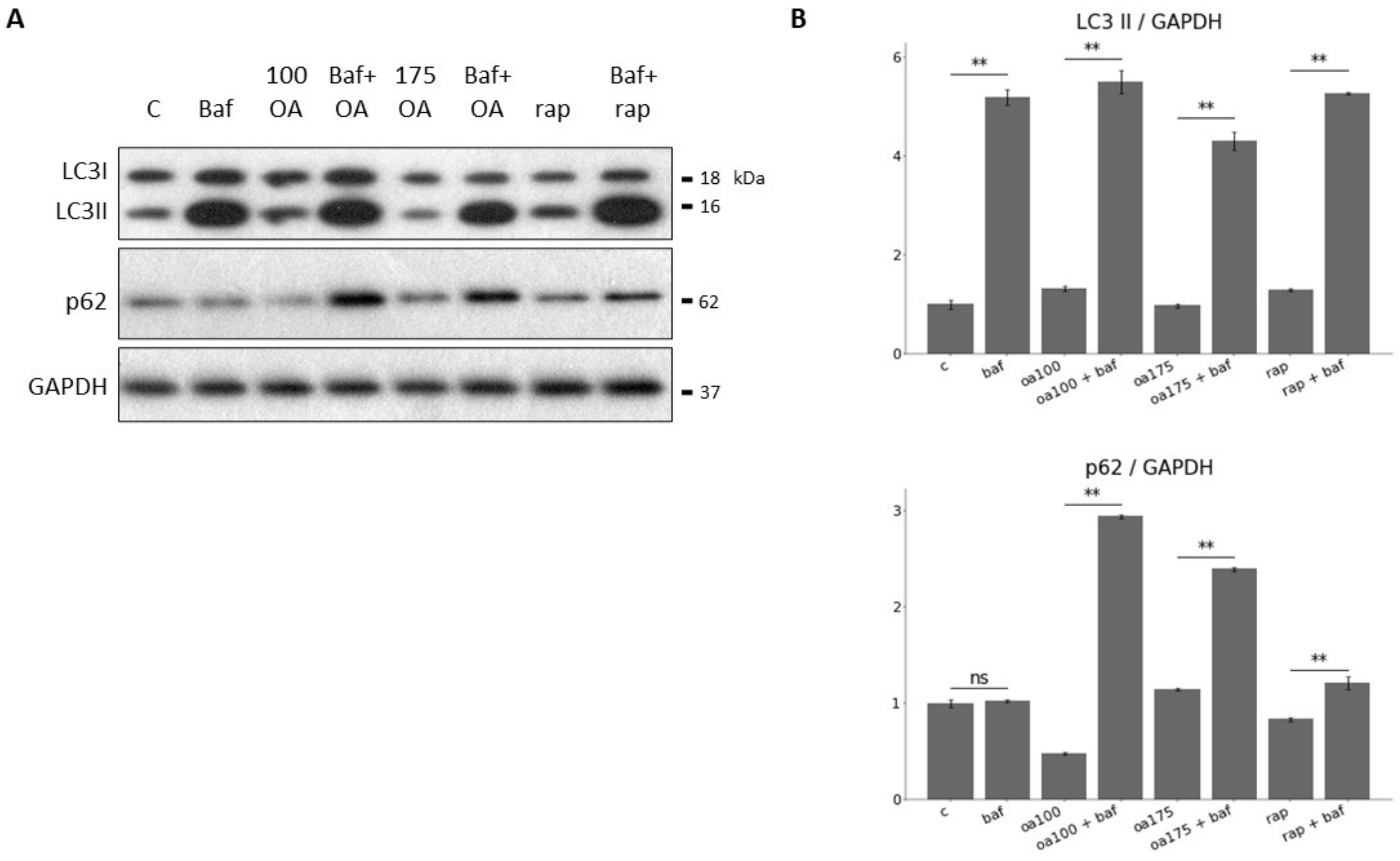

Figure S4: Analysing OA induced autophagy activation in the presence of an autophagic flux inhibitor. HEK293T cells were pre-treated without/with Bafilomycin A1 (100 nM Baf for 2 hours) followed by OA (100 nM and 175 nM for 3 hours) and rapamycin (100 nM for 2 hours). (A) The autophagy (LC3, p63) markers were followed by immunoblotting. GAPDH was used as loading control. (B) Densitometry data represent the intensity of LC3II and p62 normalized for GAPDH. For each of the experiments, three independent measurements were carried out. Error bars represent standard deviation asterisks indicate statistically significant difference from the control: ns – nonsignificant; * - $p < 0.05$; ** - $p < 0.01$.

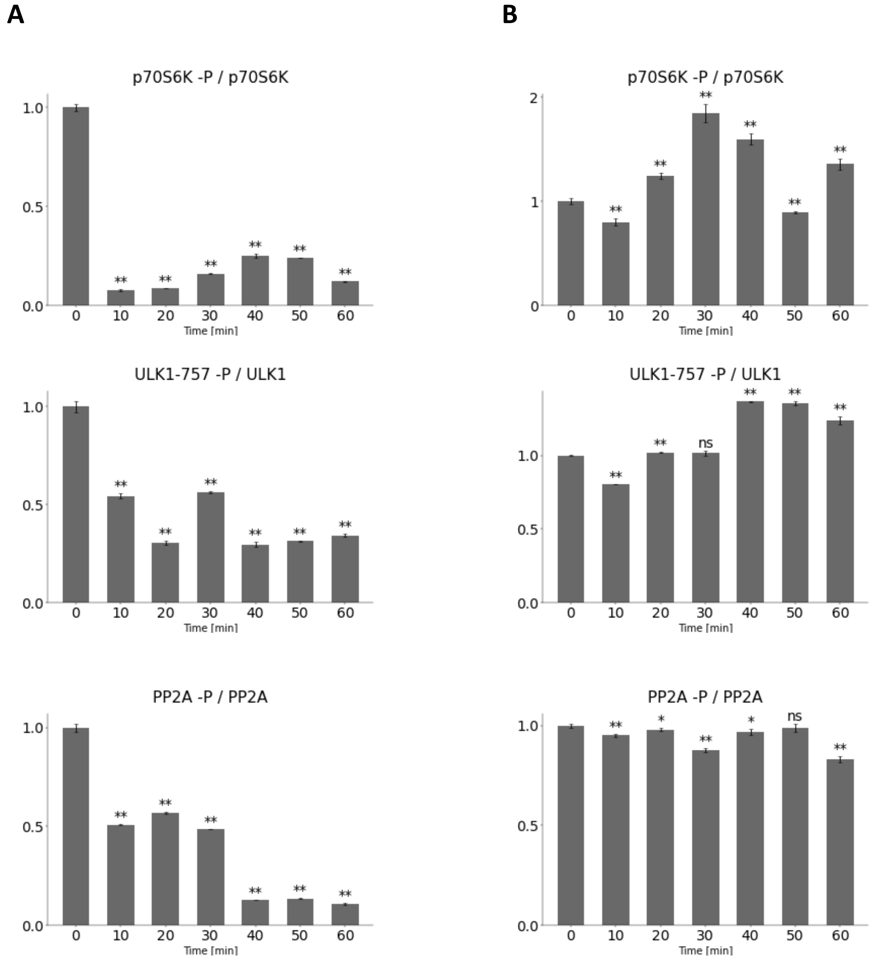

Figure S5: **A**(left) and **B**(right) show the results of the repeated quantitative analysis of the Western blot measurements shown in **Figure 2.** and **Figure 3.** respectively done with AuzureSpot Pro. For each of the experiments, three independent measurements were carried out. Error bars represent standard deviation asterisks indicate statistically significant difference from the control: ns – nonsignificant; * - p< 0.05; ** - p < 0.01.

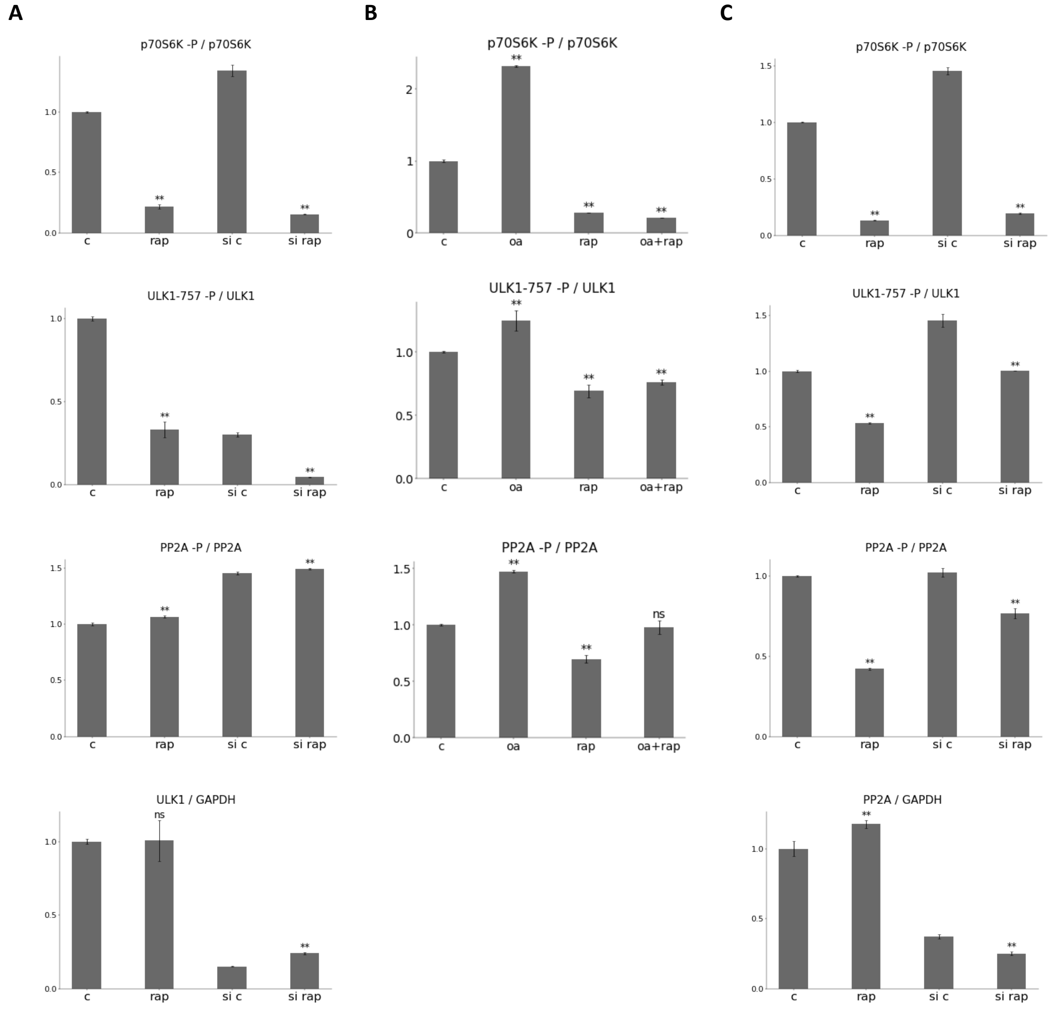

Figure S6: **A**(left), **B**(middle) and **C**(right) show the results of the repeated quantitative analysis of the Western blot measurements shown in **Figure 5/A**, **Figure 6/A** and **Figure 6/B** respectively done with AuzureSpot Pro. For each of the experiments, three independent measurements were carried out. Error bars represent standard deviation asterisks indicate statistically significant difference from the control: ns – nonsignificant; * - $p< 0.05$; ** - $p < 0.01$.

A

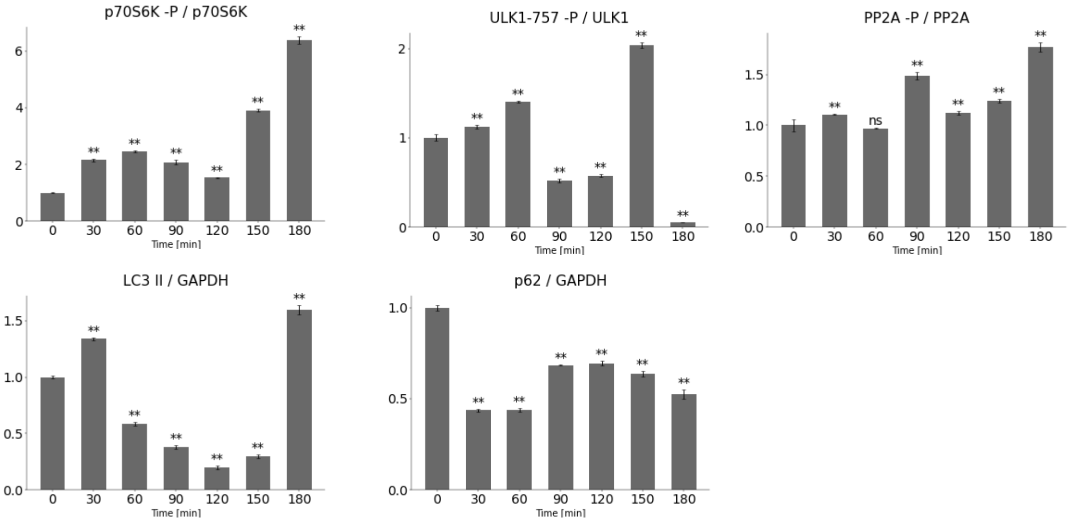

B

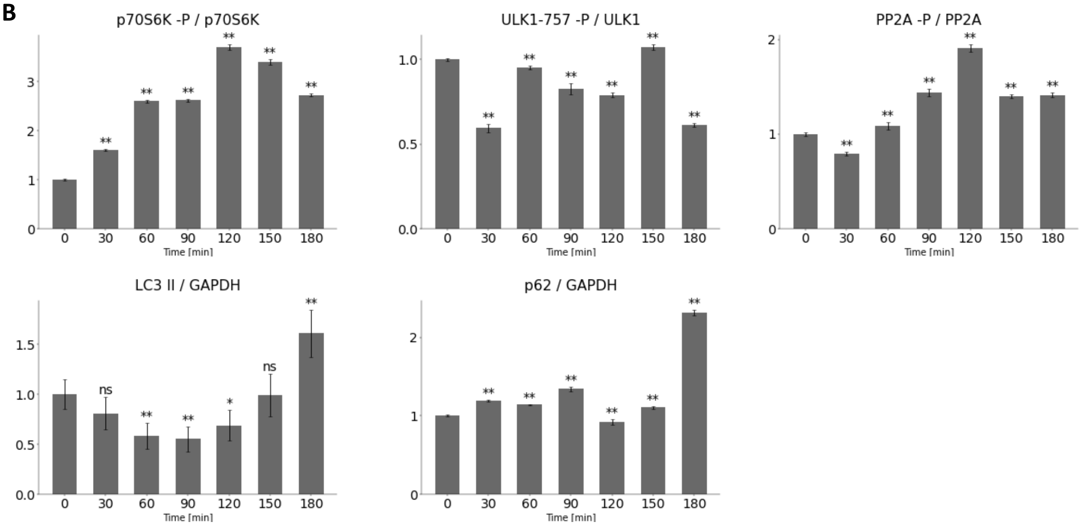

Figure S7: **A**(left), **B**(middle) and **C**(right) show the results of the repeated quantitative analysis of the Western blot measurements shown in **Figure 7/A** and **7/B.** respectively done with AuzureSpot Pro. For each of the experiments, three independent measurements were carried out. Error bars represent standard deviation asterisks indicate statistically significant difference from the control: ns – nonsignificant; * - p< 0.05; ** - p < 0.01.
